# Elucidation of the Genomic-Epigenomic Interaction Landscape of Aggressive Prostate Cancer

**DOI:** 10.1155/2021/6641429

**Published:** 2021-01-13

**Authors:** Tarun Karthik Kumar Mamidi, Jiande Wu, Chindo Hicks

**Affiliations:** ^1^Center for Computational Genomics and Data Science, Departments of Pediatrics and Pathology, University of Alabama–Birmingham School of Medicine, Birmingham, Alabama 35233, USA; ^2^Department of Genetics and the Bioinformatics and Genomics Program, Louisiana State University Health Sciences Center, School of Medicine, 533 Bolivar Street, New Orleans, LA 70112-1393, USA

## Abstract

**Background:**

Majority of prostate cancer (PCa) deaths are attributed to localized high-grade aggressive tumours which progress rapidly to metastatic disease. A critical unmet need in clinical management of PCa is discovery and characterization of the molecular drivers of aggressive tumours. The development and progression of aggressive PCa involve genetic and epigenetic alterations occurring in the germline, somatic (tumour), and epigenomes. To date, interactions between genes containing germline, somatic, and epigenetic mutations in aggressive PCa have not been characterized. The objective of this investigation was to elucidate the genomic-epigenomic interaction landscape in aggressive PCa to identify potential drivers aggressive PCa and the pathways they control. We hypothesized that aggressive PCa originates from a complex interplay between genomic (both germline and somatic mutations) and epigenomic alterations. We further hypothesized that these complex arrays of interacting genomic and epigenomic factors affect gene expression, molecular networks, and signaling pathways which in turn drive aggressive PCa.

**Methods:**

We addressed these hypotheses by performing integrative data analysis combining information on germline mutations from genome-wide association studies with somatic and epigenetic mutations from The Cancer Genome Atlas using gene expression as the intermediate phenotype.

**Results:**

The investigation revealed signatures of genes containing germline, somatic, and epigenetic mutations associated with aggressive PCa. Aberrant DNA methylation had effect on gene expression. In addition, the investigation revealed molecular networks and signalling pathways enriched for germline, somatic, and epigenetic mutations including the STAT3, PTEN, PCa, ATM, AR, and P53 signalling pathways implicated in aggressive PCa.

**Conclusions:**

The study demonstrated that integrative analysis combining diverse omics data is a powerful approach for the discovery of potential clinically actionable biomarkers, therapeutic targets, and elucidation of oncogenic interactions between genomic and epigenomic alterations in aggressive PCa.

## 1. Introduction

Prostate cancer (PCa) is the second most diagnosed and second leading cause of cancer deaths among men in the United States [1]. In 2019, an estimated 174,650 men were diagnosed with PCa and 31,620 men died from the disease [1]. Majority of the PCa deaths are attributed to localized high-grade aggressive tumours which progress rapidly to metastatic disease [2, 3]. These tumours are characterized by poor prognosis, high recurrence rates, and poor survival rates [2, 3]. The development and progression of aggressive PCa involve three separate, but related, genomes—the germline, somatic or tumour, and epigenomes [2–9]. Traditionally, the analysis of germline, somatic, and epigenetic mutations in aggressive PCa has been conducted as separate research endeavours [4]. Increasingly, germline and tumour genomes are being explored jointly to understand how genetic risk variants contribute to PCa [4]. However, to date, integration of information on germline, somatic, and epigenetic mutations to gain insights about how genetic and epigenetic mechanisms interact and cooperate to drive aggressive PCa has not been reported.

Genome-wide association studies (GWAS) have enabled discovery of germline mutations associated with an increased risk of developing PCa [4, 10]. Genetic susceptibility variants from GWAS are being incorporated in risk prediction algorithms such as polygenic risk scores (PRSs) [11, 12] to identify individual patients at the high risk of developing aggressive PCa [12–14]. PRSs are poised to improve clinical outcomes via precision medicine and precision prevention. However, one of the limitations for clinical implementation of PRSs is that the causal association between germline genetic risk variants used for calculating polygenic risk scores and aggressive PCa has not been established. Moreover, the genetic susceptibility variants reported to date explain only a small proportion of the phenotypic variation. Thus, integrating GWAS information with other omics data has the promise of not only associating genetic risk variants with tumourigenesis but also explaining the missing variation.

Advances in the next-generation sequencing technologies have enabled sequencing of the PCa or tumour and epigenomes [15, 16]. The Cancer Genome Atlas (TCGA) [15] and the International Cancer Genome Consortium (ICGC) [16] have performed large-scale sequencing of tumour and epigenomes generating vast amounts of information on somatic, epigenetic, and gene expression profiles for many cancers including PCa. However, despite the large amounts of multiomics data generated by these large cancer genome sequencing projects, genomic and epigenomic data from these projects have not been leveraged and optimally integrated with germline mutation information to elucidate the genetic-epigenetic interaction landscape in aggressive PCa. With the availability of germline, somatic, and epigenetic mutation information on PCa, we are now well-positioned to integrate these pieces of information to identify the genomic and epigenomic drivers of aggressive PCa. The objective of this investigation was to elucidate the genomic and epigenomic interaction landscape of aggressive PCa. Our working hypothesis was that aggressive PCa originates from a complex interplay between genetic (both germline and somatic mutations) and epigenomic alterations. We further hypothesized that these complex arrays of interacting genomic and epigenomic factors affect gene expression, network states, and signalling pathways which in turn drive aggressive PCa. We addressed these hypotheses using integrative data analysis combining information on germline, somatic, and epigenomic alterations using gene expression data as the intermediate phenotype. We leveraged this integrative analysis approach with network and pathway analysis to elucidate the genomic-epigenomic interaction landscape in aggressive PCa.

## 2. Materials and Methods

### 2.1. Study Design and Sources of Genomics and Epigenomics Data

**Figure 1:**
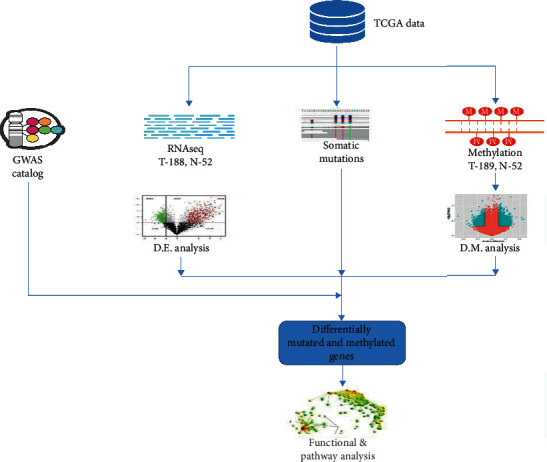
Study design and data analysis workflow for integrative analysis combining germline, somatic, and epigenetic mutation information using gene expression data as the intermediate phenotype leveraged with network and pathway analysis. TCGA: The Cancer Genome Atlas; GWAS: genome-wide association studies, T: tumours; N: normal controls; D.E.: differentially expressed; D.M.: differentially methylated. Arrows indicate the data analysis workflow.

The development and progression of aggressive PCa involve three separate, but interrelated genomes, the germline, somatic (tumour), and epigenomes. Alterations in these genomes lead to measurable changes affecting therapeutic decision-making in the in management of PCa. Therefore, the discovery of molecular drivers of aggressive PCa should take a comprehensive approach that combines pieces of information from all three genomes. Here, we used an integrative genomics approach that combines germline mutation from GWAS with somatic mutation and DNA methylation from TCGA using gene expression data as the intermediate phenotypes and unifying parameter. The integrative analysis approach was leveraged with network and pathway analysis to elucidate possible oncogenic interactions between genes containing germline, somatic, and epigenetic mutations. The overall project design showing sources of data and analysis workflow integrating multiomics data is shown in Figure 1.

Germline mutation data was obtained from a well-curated and annotated catalogue of genetic variants associated with an increased risk of developing PCa that we have developed and published [4, 17]. Details pertaining data collection, curation, and annotation have been published elsewhere [4, 17] and were based on international guidelines for assessing cumulative evidence on GWAS associations [18–22]. This data was supplemented with data from the updated GWAS catalogue [10, 23, 24]. Overall, the GWAS data set included 401 genes containing 631 germline mutations (single-nucleotide polymorphisms (SNPs)) associated with an increased risk of developing PCa, linked with SNP identification numbers (rs-IDs), evidence of association as determined by the GWAS *P* value, gene name, and associated chromosome position. Information on SNP-IDs and gene names was further verified using the single-nucleotide polymorphisms database (dbSNP) (https://www.ncbi.nlm.nih.gov/snp/) [25] and the Human Genome Nomenclature Committee (HGNC) database (https://www.genenames.org/) which houses approved gene names and their aliases [26]. Information on genes and germline mutations including the original reports from which the information was derived is presented in Supplementary Table S1.

Somatic mutation information, DNA methylation, and gene expression along with clinical variables on aggressive PCa were obtained from The Cancer Genome Atlas (TCGA) [27]. The data were downloaded from the Genomics Data Commons portal (https://portal.gdc.cancer.gov/) using the data transfer tool [28]. Somatic mutation, DNA methylation, and gene expression were all generated on the same 188 individual patients diagnosed with aggressive PCa and 52 control samples. All the samples were linked with clinical information. Aggressive tumours were defined as tumours with Gleason grade 8-10 and or Gleason grade 7 with pathological score of 4 + 3 (primary + secondary) and were authenticated using clinical information and the American Urological Association (AUA) protocol [29]. Gene expression data was checked for quality by removing the genes (rows) with missing data, such that each row had at least ≥30% data using counts per million (CPM) filter (>0.5) implemented in R [30]. The resulting data set with 18,428 probes was normalized using the trimmed mean of *M* value (TMM) normalization method and transformed using Voom module in the Limma package implemented in R [30]. Probe IDs were replaced by annotated gene symbols and names using the Ensemble database. Somatic mutation data was processed to identify the number of genes containing somatic mutations and the number of somatic mutations per gene across samples. This processing step generated a catalogue of 4,779 somatic mutated genes and 6,658 somatic mutation events used in the analysis. A complete list of somatic mutated genes and number of somatic mutation events per gene is presented in Supplementary Table S2.

As noted, DNA methylation data was generated from the same 188 tumour and 52 control samples as gene expression and somatic mutation data using the Illumina HumanMethylation450 BeadChip [31]. The data was processed using the Illumina DNA methylation data processing and analysis protocols [32, 33] implemented in our pipeline [34]. The data was corrected for batch effects and normalized using quantile normalization implemented in the R Package consistent with Illumina DNA methylation data analysis protocol [31–35].

### 2.2. Bioinformatics Analysis

We performed gene expression and DNA methylation data analysis using the pipelines we have developed and implemented in R Bioconductor packages [34]. We performed whole transcriptome analysis comparing gene expression levels between tumour and control samples using the Limma package implemented in R [30] to identify all significant differentially expressed genes distinguishing aggressive tumours from control samples. We used the false discovery rate (FDR) procedure to control for multiple hypothesis testing [36]. Genes were ranked on *P* values, log2 fold change (LogFC), and FDR. Likewise, we performed whole methylome analysis comparing DNA methylation profiles between tumours and control samples to discover a signature of significantly differentially methylated genes and CpG sites using the Limma package implemented in R [30]. We employed the FDR in the analysis to correct for multiple hypothesis testing [36]. The discovered CpG sites were annotated with gene symbols using the Ensemble Biomart database [37]. We computed the number of CpG sites per gene for significantly differentially methylated genes to get a quantitative assessment of DNA methylation sites per gene. The methylation sites were classified as either hypomethylated (down) or hypermethylated (up) based on the direction of regulation using the Limma package [30]. The genes and CpG sites were then ranked on *P* values, LogFC, FDR, and number of significantly (*P* < 0.05) differentially methylated sites. Differentially expressed genes and differentially methylated genes were merged and sorted by gene symbols, expression, and methylation *P* values to discover a signature of differentially expressed genes which were also differentially methylated. We investigated the impact of DNA methylation on gene expression using a two-way plot of expression LogFC against the DNA methylation LogFC using the Starburst plot [38] using only differentially expressed genes which were also differentially methylated. Genes associated with the diseases were further evaluated for the presence of germline and somatic mutations to identify a signature of genes containing germline, somatic, and epigenetic mutations transcriptionally associated with aggressive PCa. Genes containing germline, somatic, and epigenetic alterations associated with aggressive PCa were subjected to network and pathways analysis using Ingenuity Pathway Analysis (IPA) software package [39] to identify gene regulatory networks and signalling pathways enriched for the three types of mutations. We used gene ontology (GO) [40] analysis implemented in IPA to characterize the genes according to molecular function, biological processes, and cellular components in which they are involved.

## 3. Results

### 3.1. Discovery of Gene Expression and DNA Methylation Signatures

**Figure 2:**
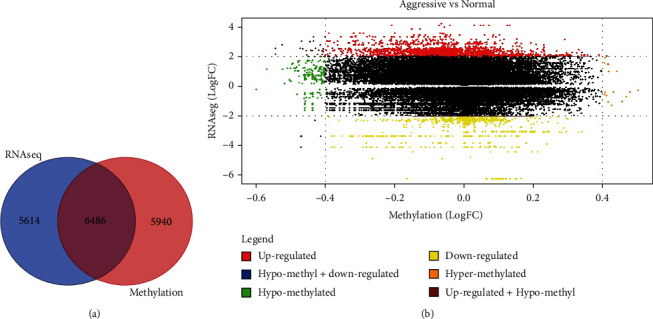
(a) Distribution of significantly (*P* < 0.05) differentially methylated and differentially expressed genes (intersection shows the 6,486 significantly differentially expressed genes which were also significantly differentially methylated). (b) Shows a two-way Starburst plot of differentially expressed genes (*y*-axis) and differentially methylated genes (*x*-axis) along with their direction of change indicated by the colour code key. The colour codes (key) indicate the direction of change for the genes under study.

To discover gene expression and DNA methylation signatures associated with aggressive PCa, we performed whole methylome and whole transcriptome analysis comparing tumour to control samples separately. The results of this investigation are summarized in Figure 2(a). The comparison of DNA methylation profiles between tumour and control samples revealed a signature of 12,426 significantly (*P* < 0.05) differentially methylated genes associated with aggressive PCa (Figure 2(a)). There was significant variation in patterns of DNA methylation profiles and the number of CpG sites associated with aggressive PCa.

The number of CpG sites per gene ranged from 1 to 480 in tumour samples. The most highly significantly differentially methylated genes were *PTPRN2*, *PRDM16*, *PCDHGA1*, *PCDHGA2*, *PCDHGA3*, *PCDHGB1*, *MAD1L1*, *PCDHGA4*, *PCDHGB2*, *PCDHGA5*, *PCDHGB3*, and *PCDHGA6* with ≥200 significantly (*P* < 0.05) differentially methylated CpG sites per gene. A complete list of all significantly differentially methylated genes distinguishing tumour samples from controls along with the number of differentially methylated CpG sites per gene is presented in Supplementary Table S3. The comparison of gene expression levels between tumour and control samples produced a signature of 12,100 significantly (*P* < 0.05) differentially expressed genes (Figure 2(a)). The most highly significantly differentially expressed genes were *SIM2*, *HOXC6*, *NKX2-3*, *DLX1*, *EPHA10*, *PCAT7*, *ARHGEF38*, *PRR36*, and *EZH2* (*P* < 10^−11^). A complete list of significantly differentially expressed genes associated with aggressive PCa is presented in Supplementary Table S4.

To address the hypothesis that aberrantly expressed genes associated with aggressive PCa are also aberrantly expressed, we combined the 12,426 significantly (*P* < 0.05) differentially methylated genes with the 12,100 significantly (*P* < 0.05) differentially expressed genes and ranked the genes based on expression and CpG sites *P* values. The analysis produced a signature of 6,486 containing both alterations (Figure 2(a), intersection). In addition, the investigation produced a signature of 5,614 genes altered in the transcriptome only and a signature of 5,940 genes with only epigenetic alterations associated with aggressive PCa (Figure 2(a)). The discovery of a signature of genes altered in both the trascriptome and the methylome and signatures of different sets of genes altered in each of them demonstrates the power of integrative analysis using complementary technologies.

Having discovered the 6,486 aberrantly methylated genes transcriptionally associated with aggressive PCa (Figure 2(a)), we conducted additional investigation on these genes to determine whether DNA methylation affects gene expression. The results showing the effect of aberrant DNA methylation on gene expression are presented in a two-way Starburst plot in Figure 2(b). The investigation revealed that aberrant DNA methylation affects gene expression (Figure 2(b)). We discovered 206 upregulated, 77 down regulated, 152 hypomethylated, and 30 hypermethylated genes (Figure 2(b)). Three genes *HOXC4*, *HOXC6*, and *NOX4* were hypomethylated and downregulated, whereas 14 genes *CYP27A1*, *NRK*, *EMX2OS*, *C2orf88*, *PRKCB*, *WFDC2*, *NRG2*, *MCF2*, *COL4A6*, *PROM1*, *AOX1*, *HIF3A*, *CYP11A1*, and *GATA3* were hypomethylated and upregulated. The gene *SLC2A9* was hypermethylated and upregulated. The results confirmed our hypothesis that aberrant DNA methylation affects gene expression at varying levels.

To determine the extent of epigenomic alterations for the 6,486 genes containing both alterations, we computed the *P* values for the most variable CpG sites and the number of CpG sites across tumour samples for each gene. Genes were ranked according to the number of CpG sites in the gene. The results showing the top 23 most highly significantly differentially methylated genes with >100 CpG sites per gene are presented in Table 1. Also presented in the table are probes showing the most highly significant CpG sites, their estimates of *P* values, number of CpG sites per gene, and estimates of gene expression p-values.

The analysis revealed significant variation in patterns of DNA methylation profiles among the genes (Table 1). The number of CpG sites per gene ranged from 1 to 480. The genes *PTPRN2*, *PRDM16*, *PCDHGA1*, *PCDHGA2*, *PCDHGB1*, *MAD1L1*, *PCDHGA4*, *PCDHGB2*, *PCDHGA5*, *PCDHGB3*, *PCDHGA6*, *RPTOR*, *COL11A2*, *KCNQ1*, *PCDHA1*, *PCDHGA9*, *PCDHGB6*, *AGAP1*, *ATP11A*, *PCDHGA10*, *PCDHGB7*, *MCF2L*, and *CACNA1HA* had the most highly significantly differentially CpG sites and the highest number of CpG sites per gene ≥ 100 CpG sites (Table 1). Among the 23 genes in Table 1 included the genes *PTPRN2*, *PCDHGB1*, *ATP11A*, and *CACNA1HA* which have been experimentally confirmed to be associated with aggressive PCa [41–44]. A complete list of all the 6,486 genes containing both genomic and epigenomic alterations along with the number of methylation sites per gene is presented in Supplementary Table S5. Taken together, the results of these investigations show that a subset of genes that are transcriptionally associated with tumours is aberrantly methylated and that aberrantly methylated genes affect gene expression in aggressive PCa.

### 3.2. Discovery of Somatic Mutation and DNA Methylation Signatures

Although development and progression of aggressive PCa tumours are driven by acquired somatic driver mutations [3], enduring epigenetic landmarks define the tumour microenvironment [45]. Therefore, our next step in this investigation was to determine whether aberrantly methylated genes transcriptionally associated with aggressive PCa are somatic mutated. We hypothesized that aberrantly methylated genes transcriptionally associated with aggressive PCa are somatic mutated. We addressed this hypothesis by integrating somatic mutation information with epigenomic and gene expression data. Specifically, we evaluated aberrantly methylated genes transcriptionally associated with aggressive PCa for the presence of somatic mutations using the 4,779 genes containing somatic mutations.

**Figure 3:**
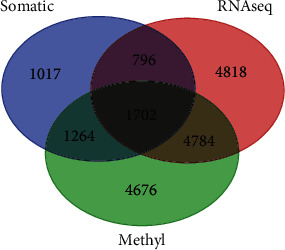
Three-way Venn diagram showing the results of somatic mutated, aberrantly DNA methylated, differentially expressed genes associated with aggressive PCa discovered through analysis and integration of somatic mutation, DNA methylation, and gene expression data.

The results of this investigation are presented in a three-way Venn diagram shown in Figure 3. The analysis revealed a signature of 1,702 genes containing all three alterations (Figure 3). In addition, the analysis produced a signature of 796 somatic mutated genes transcriptionally associated with the disease and a signature of 1,264 somatic mutated aberrantly methylated in aggressive PCa (Figure 3). A total of 1,017 somatic mutated genes were neither aberrantly methylated nor transcriptionally associated with the disease (Figure 3). A complete list of all the 1,702 somatic mutated genes aberrantly methylated and transcriptionally associated with aggressive tumours is presented in Supplementary Table S6. A complete list of the 796 somatic mutated genes transcriptionally associated with the diseases and a complete list of the 1,264 somatic mutated genes aberrantly methylated in aggressive PCa are presented in Supplementary Table S7.

To determine the extent of somatic and epigenetic alterations and whether the most highly mutated genes are the most highly epigenetically altered and or vice versa, we evaluated the 1,702 genes containing all three alterations (Figure 3). The results showing the top 45 most highly somatic mutated (>3 somatic events per gene) genes are presented in Table 2. Also presented in Table 2 are the most highly significant CpG sites and associated *P* values along with the number of CpG sites per gene and gene expression *P* values.

There was significant variation in the distribution of somatic mutations and methylation sites per gene. The most highly somatic mutated genes were *SPOP*, *FOXA1*, *LRP1B*, *OBSCN*, *CSMD3*, *FREM2*, *AHNAK*, *PLCB4*, *SYNE1*, *PCDH18*, *CDH23*, *DCHS2*, *VPS13D*, *MACF1*, *PTPRD*, *HFM1*, *AHNAK2*, *CTNNB1*, and *SACS* (Table 2). Further evaluation of the results revealed that not all highly somatic mutated genes were highly differentially methylated (Table 2). The most highly differentially methylated genes were *SPOP*, *OBSCN*, *CSMD3*, *AHNAK*, *SYNE1*, *CDH23*, *DCHS2*, *VPS13D*, *MACF1*, *PTPRD*, *TACC2*, *GRIN2A*, *PCDHGA9*, *SALL1*, *NPAT*, *DST*, *CACNA1C*, *ZFHX3*, *PCDHA1*, *EPHA3*, and *PTEN* (Table 2). Conversely, not all the most highly somatic mutated genes were highly differentially methylated. The observed significant variation in DNA methylation can be explained in part by the phenotypic heterogeneity inherent in aggressive PCa [8]. Overall, the investigation revealed that a subset of aberrantly methylated genes is somatic mutated and that the distribution of somatic and epigenetic alterations in these genes varies significantly. The discovery of somatic mutated genes which were also epigenetically altered suggests that some of the genes driving tumourigenesis may be under genetic and epigenetic control.

### 3.3. Discovery of Germline, Somatic, and Epigenetic Mutation Signatures

As noted earlier in this report and consistent with other reports [2–9], the development and progression of aggressive PCa involve three separate, but related, genomes—the germline, somatic or tumour, and epigenomes. Therefore, optimal integration of omics data should include all three genomes and the phenotype they regulate. Thus, to address the hypothesis that somatic and epigenetics mutated genes associated with aggressive PCa harbour germline mutations and to infer the potential causal association between genetic susceptibility and aggressive PCa, we evaluated the 401 genes containing germline mutations for their association with aggressive PCa using gene expression information and for the presence of somatic mutations and epigenetic alterations.

**Figure 4:**
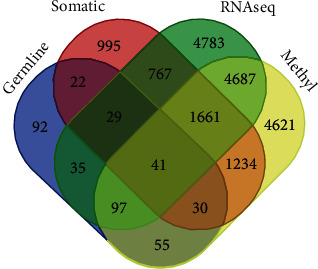
Four-way Venn diagram showing signatures of genes containing germline, somatic, and epigenetic mutations transcriptionally associated with aggressive PCa discovered through analysis and integration germline and somatic mutation, DNA methylation, and gene expression data.

The results of this investigation are presented in a four-way Venn diagram in Figure 4. Out of the 401 genes containing germline mutations evaluated, 41 genes contained germline, somatic, and epigenetic alterations and were transcriptionally associated with aggressive tumours. In addition, we discovered 202 genes transcriptionally associated with aggressive PCa, 223 genes aberrantly methylated, 122 genes somatic mutated, and 97 aberrantly methylated genes transcriptionally associated with the disease (Figure 4). A subset of 92 genes was altered only in the germline and was neither aberrantly methylated nor transcriptionally associated with the disease (Figure 4). Overall, the investigation confirmed our hypothesis that genes containing germline mutations are associated with aggressive PCa and harbour both somatic and epigenetic alterations. The discovery of genes altered only in the germline can be explained partially by the differences in population cohorts from which GWAS and sequence data were derived. GWAS discoveries are inherently heterogeneous and derived from heterogeneous populations, which gene expression can be population and time specific. Under such conditions, the observed outcome is expected.

In addition to evaluating the distribution of genes containing germline, somatic, and epigenetic mutations, we performed a quantitative assessment on the discovered gene signatures to evaluate the frequency distribution and extent of germline, somatic, and epigenetic mutation events among the 41 genes containing all three alterations. The results of this investigation are presented in Table 3.

There was significant variation in the distribution of germline, somatic, and epigenomic alterations (Table 3). The number of somatic and germline mutations was lower than the number of CpG sites in each gene (Table 3). Interestingly, the 41 gene signature included the genes *BRCA1*, *KLK3*, *KLK2*, *PDLIM5*, and *ITGA6*, containing genetic variants reported to be directly associated with aggressive PCa [4, 46–48], and the genes *AMIGO2*, *ATF71P*, *BRCA1*, *KLK2*, *KLK3*, *MDM4*, and *PDLIM5* used in gene panels for PCa screening and assessing disease prognosis [46–48]. Overall, the investigation confirmed our hypothesis that somatic and epigenetic mutated genes harbour germline mutations and provides some foundational knowledge about the potential link between the genetic susceptibility variants and tumourigenesis. The discovery of epigenetic mutated genes without germline mutations tends to suggest that part of the missing variation not explained by GWAS may be explained by DNA methylation.

### 3.4. Discovery of Altered Molecular Networks and Signalling Pathways

The objective of this investigation was to elucidate the genomic and epigenomic interaction landscape of aggressive PCa. The results in preceding sections have shown that genes genetically altered in the tumour genome are aberrantly methylated and that somatic and epigenetic mutated genes harbour germline mutations. To gain insights about the possible oncogenetic interactions between genetic and epigenetic changes, we performed network and pathway analysis. Our working hypothesis was that aggressive PCa originates from a complex interplay between genomic (involving both germline and somatic mutations) and epigenomic alterations. We further hypothesized that these complex arrays of interacting genomic and epigenomic factors affect gene expression, molecular networks, and signalling pathways which in turn drive aggressive PCa. We addressed these hypotheses using network and pathways analyses to identify molecular networks and signalling pathways enriched for genetic and epigenetic alterations and characterized their functional connectivity. For this analysis, we used the 41 genes containing germline, somatic, and epigenetic mutations. Because genes containing germline mutations explain only a small proportion of the phenotypic variation and their causal association with the disease has not been established, we also included the most highly somatic and epigenetic mutated genes without germline mutations.

**Figure 5:**
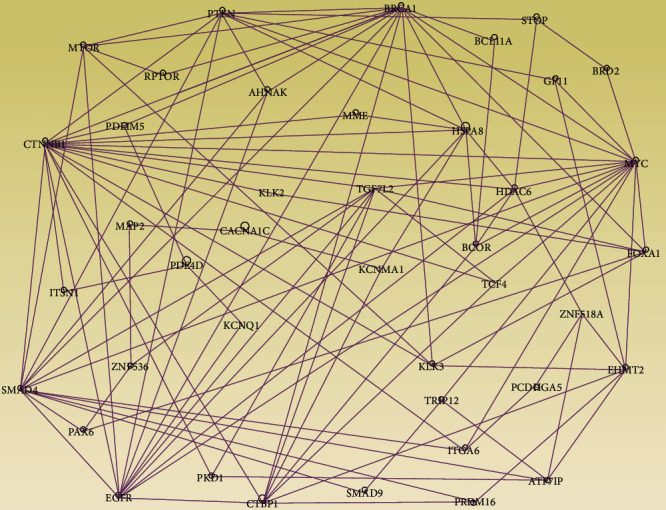
Molecular networks enriched for germline, somatic, and epigenomic mutations. The nodes represent the genes in gene symbols, and vertices represent functional relationships. Genes in blue fonts contain germline, somatic, and epigenetic mutations. Genes in red fonts contain somatic and epigenetic mutations. Genes in green fonts are highly differentially methylated genes with greater than 50 DNA methylation sites per gene.

The results of network analysis are presented in Figure 5. Network analysis produced 19 molecular networks with the *Z*-scores ranging from 2 to 51. The analysis revealed functionally related genes containing germline, somatic, and epigenomic alterations interacting in gene regulatory networks (Figure 5).

The discovered networks contained genes predicted to be involved in cancer, cell-to-cell signalling and interaction, organismal injury and abnormalities, reproductive system disease, cellular assembly and organization, amino acid metabolism, posttranslational modification, immunological disease, DNA damage and repair, and hereditary disorder. The analysis also produced molecular networks containing genes predicted to be involved in cell cycle, cell death and survival, cellular development, organ development, and reproductive system development and function. Among the genes revealed by network analysis included the genes *KLK3*, *ITGA6*, and *BRCA1* containing germline mutations directly associated with aggressive cancer Figure 5 [4]. Overall, the investigation revealed molecular networks enriched for germline, somatic, and epigenetic mutations involved in aggressive PCa. The investigation confirmed our working hypothesis was that aggressive PCa is an emergent property of molecular networks of functionally related genes containing germline, somatic mutations, and epigenetic alterations.

**Figure 6:**
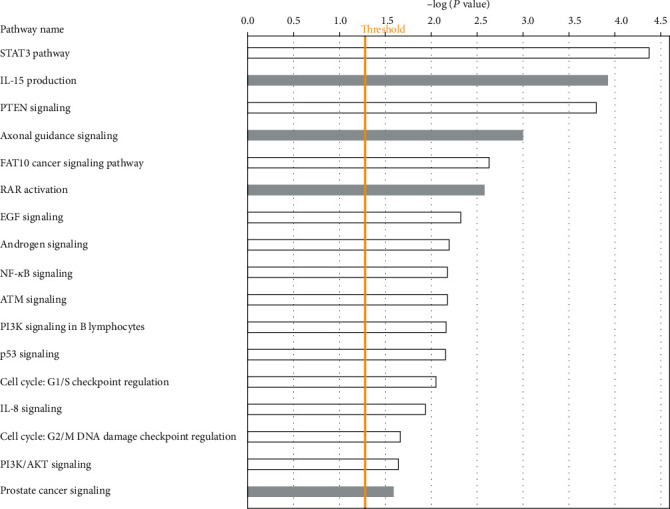
Signalling pathways enriched for germline, somatic, and epigenetic mutations in aggressive PCa. The *y*-axis shows the pathway names, and the *x*-axis shows the –log(*P* values) on which pathways were ranked and selected. The yellow line indicates the threshold level expressed as the –log(*P*-value) above which the signalling pathway was declared significant.

Pathway analysis revealed 96 signalling pathways enriched for germline, somatic, and epigenetic mutations. The topmost highly significant signalling pathways are presented in Figure 6. Also presented in the figure is the threshold *P* value marked by the yellow line, above which the pathways were declared significant following correction for multiple hypothesis testing. The investigation revealed the STAT3, IL-15, PTEN, axonal guidance, cancer, FAT10 cancer, RAR activation, EGF, androgen, NF-*κ*B, ATM, PI3K, and P53 signalling pathways (Figure 6). In addition, the investigation revealed the cell cycle: G1/S checkpoint regulation, and IL-8; and cell cycle: G2/M DNA damage checkpoint regulation, PI3K/AKT, and the PCa signalling pathways (Figure 6). Overall, the results of the investigation confirmed our working hypothesis that oncogenic interactions among genes containing genetic and epigenetic mutations affect signalling pathways which in turn drive aggressive PCa.

In summary this integrative data approach combining multi-omics data revealed that genomic and epigenomics alterations in the germline and tumour genomes can lead to measurable changes that could guide elucidation of the genomic-epigenomic landscape in aggressive PCa. This interdisciplinary integrated approach establishes putative functional bridges between germline, somatic (tumour), and epigenetics and the pathways the control. These observations suggest that genes and pathways driving aggressive PCa are under genetic and epigenetic control and that integrative analysis combining data from complementary technologies provides a unified and optimal approach to the discovery of potential clinically actionable biomarkers and targets for the development of novel therapeutics in aggressive PCa.

## 4. Discussion

The last decade has witnessed remarkable progress in the discovery and development of comprehensive catalogues of germline genetic susceptibility variants associated with an increased risk of developing PCa using GWAS [4, 5, 10, 17]. In parallel to large-scale genotyping, next-generation sequencing has generated massive amounts of genomic and epigenomic data from tumour genomes [15, 16]. Traditionally, genotyping and sequencing have been conducted as separate research endeavours. Here, we combined information on germline, somatic, and epigenetic alterations using gene expression data as the intermediate phenotype to elucidate the genomic-epigenomic interaction landscape of aggressive PCa. The investigation revealed functionally related germline, somatic, and epigenetic mutated genes associated with aggressive tumours. The investigation further revealed molecular networks and signalling pathways enriched for genetic and epigenetic mutations and that DNA methylation affects gene expression. To the best of our knowledge, this is the first study to comprehensively integrate information on germline, somatic, and epigenetic mutations at the gene, network, and pathway levels using gene expression as the intermediate phenotype. We summarize the clinical significance and translational aspects of this investigation as follows.

First, the discovery of genes such as *KLK3* and *AR* altered in germline, somatic (tumour), epigenome, and the transcriptome, coupled with the findings that aberrant DNA methylation affects gene expression demonstrates that integrative analysis combining information from complimentary technologies provides a unified approach for the discovery of potential clinically actionable biomarkers in aggressive PCa. Indeed, aberrant DNA methylation in PCa has been reported [49–51]. The novel and innovative aspects of our investigation are that they combine diverse omics data and assesses the impact of DNA methylation on gene expression and to establish putative functional bridges between germline, somatic, and epigenetic alterations and the pathways they control in aggressive PCa.

Second, the discovery of genes such as *BRCA1*, *AR*, *ATM*, and *KLK3* containing germline, somatic, and epigenetic mutations is of particular interest. This reveals a potential link between genetic susceptibility and tumourigenesis. Importantly, while tumour development and progression may be driven by acquired somatic driver mutations in these genes, the actions of somatic mutations maybe primed by germline mutations and enduring epigenetic landmarks may be defining the tumour microenvironment [45]. Moreover, epigenetic alterations in DNA repair genes such as *BRCA1* and *ATM* discovered in this investigation could cause genome instability and silencing of tumour suppressor genes, such as *P53*, leading to carcinogenesis [52–54].

Third, the discovery of a signature of 41 genes containing germline, somatic, and epigenetic alterations is of particular interest. To date, risk prediction algorithms such as PRSs use germline mutations mapped to genes used in this investigation [11–14]. However, the causal association between genetic susceptibility variants used in computing PRSs and aggressive PCa has not been established. Moreover, the genetic susceptibility variants reported thus far explain only a small proportion of the phenotypic variation, which raises the question of “where is the missing heritability”?. Incorporation of somatic mutation, epigenetic, and gene expression data as demonstrated here has the potential to address some of the limitations incurred in current risk prediction models and could address the question of missing variation not accounted for by risk variants [55, 56]. This could be achieved by leveraging germline mutation information and integrating it with somatic and epigenetic mutation using gene expression data as demonstrated here to develop more robust and more accurate genetic risk prediction models to enhance precision medicine and precision prevention [57]. This is an attractive approach because both germline and epigenomic variations are heritable and affect gene expression variation [58–60].

Fourth, the discovery of key signalling pathways implicated in aggressive PCa including STAT3, PTEN, molecular mechanisms of cancer, AR, ATM, PI3K/AKT, PCa, and P53 signalling pathways [61, 62] was intriguing. First, it demonstrates that the signalling pathways driving aggressive PCa are likely under genetic and epigenetic control. Second and perhaps more importantly is that these findings provide a rational basis for the discovery of potential targets critical to the development of novel therapeutics for aggressive PCa. This is noteworthy because, currently, the AR and PI3K signalling pathways are used as therapeutic targets in aggressive PCa, as androgen-deprivation therapy (ADT) is one of the most effective therapeutic modalities [61, 62]. Overall, this comprehensive multidisciplinary approach to elucidation of the genomic-epigenomic interaction landscape of aggressive PCa provides novel insights about the power of integrative analysis combining diverse omics data for the discovery of genetic and epigenetic drivers of aggressive PCa and how they interact and cooperate to drive the clinical phenotypes.

## 5. Conclusions

The investigation revealed DNA methylation and gene expression signatures associated with aggressive PCa and that aberrant DNA methylation affects gene expression. The investigation revealed that germline and somatic mutated genes are aberrantly methylated and transcriptionally associated with aggressive PCa. The investigation revealed that aggressive PCa is an emergence property of gene regulatory networks and signalling pathways under genetic and epigenetic controls. Integrative analysis combining genomic and epigenomic data using gene expression as the intermediate phenotype is a powerful approach for elucidating the genomic-epigenomic interaction landscape in aggressive PCa, discovery of potential clinically actionable biomarkers, and targets for the development of novel therapeutics.

## Figures and Tables

**Table 1:** List of the 23 most highly significantly differentially methylated genes with greater than 100 CpG sites per gene which were also differentially expressed associated with aggressive PCa. Probe and associated *P* value indicate the most significant CpG site.

Gene symbol	Cytoband	Methylation			RNA-Seq
		CpG probes	*P* values	CpG sites	*P* values
*PTPRN2*	7q36.3	cg27448110	1.13*E* − 30	480	0.00018
*PRDM16*	1p36.32	ch.1.131529R	6.00*E* − 06	472	3.48*E* − 10
*PCDHGA1*	5q31	cg27665767	1.25*E* − 08	294	2.00*E* − 12
*PCDHGA2*	5q31.3	cg27665767	1.25*E* − 08	288	0.048678
*PCDHGB1*	5q31	cg27665767	1.25*E* − 08	264	4.76*E* − 10
*MAD1L1*	7p22.3	ch.7.111787F	0.006761	261	0.000725
*PCDHGA4*	5q31	cg27665767	1.25*E* − 08	258	0.034977
*PCDHGB2*	5q31	cg27665767	1.25*E* − 08	243	1.04*E* − 05
*PCDHGA5*	5q31	cg27665767	1.25*E* − 08	236	3.28*E* − 06
*PCDHGB3*	5q31	cg27665767	1.25*E* − 08	221	0.01935
*PCDHGA6*	5q31	cg27665767	1.25*E* − 08	211	0.002877
*RPTOR*	17q25.3	cg27511181	0.030313	185	3.13*E* − 09
*COL11A2*	6p21.32	cg27590742	7.21*E* − 06	178	0.001095
*KCNQ1*	11p15.5	cg27639104	0.036658	160	1.15*E* − 07
*PCDHA1*	5q31.3	cg27604145	0.000777	147	1.86*E* − 05
*PCDHGA9*	5q31	cg27639030	4.84*E* − 07	145	6.14*E* − 07
*PCDHGB6*	5q31	cg27639030	4.84*E* − 07	136	0.006861
*AGAP1*	2q37.2	cg27634020	2.03*E* − 06	134	1.04*E* − 07
*ATP11A*	13q34	cg27096043	1.07*E* − 09	127	7.25*E* − 05
*PCDHGA10*	5q31.3	cg27639030	4.84*E* − 07	124	0.013509
*PCDHGB7*	5q31.3	cg27639030	4.84*E* − 07	109	8.81*E* − 05
*MCF2L*	13q34	cg27359668	0.031441	105	1.46*E* − 10
*CACNA1H*	16p13.3	cg27616039	1.24*E* − 16	103	1.64*E* − 06

**Table 2:** List of the top 45 genes containing both somatic and epigenetic mutation with greater than 3 somatic mutations and number of CpG sites per gene along with estimates of differential gene expression and DNA methylation probe *P* values.

*Genes*	Cytoband	Methylation			RNAseq	
		Probes	Adjusted *P* value	CpG sites	Adjusted *P* value	Somatic mutations
*SPOP*	17q21.33	cg14245135	1.69*E* − 60	10	4.31*E* − 23	29
*FOXA1*	14q21.1	cg01824511	3.51*E* − 23	1	2.24*E* − 30	12
*LRP1B*	2q22.1	cg21484213	1.17*E* − 09	5	1.11*E* − 17	10
*OBSCN*	1q42.13	cg05794117	2.11*E* − 32	57	0.000501	9
*CSMD3*	8q23.3	cg22433418	1.82*E* − 43	31	0.026425	8
*FREM2*	13q13.3	cg24087887	1.53*E* − 05	2	3.57*E* − 06	8
*AHNAK*	11q12.3	cg05427381	1.67*E* − 28	14	0.00191	7
*PLCB4*	20p12.3	cg09143713	2.34*E* − 21	6	9.80*E* − 07	7
*SYNE1*	6q25.2	cg11318342	1.42*E* − 20	46	2.38*E* − 05	7
*PCDH18*	4q28.3	cg12033966	0.019046	2	3.03*E* − 09	7
*CDH23*	10q22.1	cg24331301	7.70*E* − 56	40	1.35*E* − 19	6
*DCHS2*	4q31.3	cg00067274	7.70*E* − 37	18	1.97*E* − 17	6
*VPS13D*	1p36.22	cg20931951	1.58*E* − 33	35	0.006686	6
*MACF1*	1p34.3	cg14713026	1.56*E* − 27	16	0.010637	6
*PTPRD*	9p24.1	cg14258031	1.12*E* − 17	17	0.034734	6
*HFM1*	1p22.2	cg25188594	6.08*E* − 11	3	0.008453	6
*AHNAK2*	14q32.33	cg06903818	3.01*E* − 28	1	2.92*E* − 17	5
*CTNNB1*	3p22.1	cg05726118	6.92*E* − 16	6	0.023068	5
*SACS*	13q12.12	cg18653350	8.60*E* − 12	1	0.000469	5
*TACC2*	10q26.13	cg06733794	2.09*E* − 58	37	0.023942	4
*GRIN2A*	16p13.2	cg01348055	1.07*E* − 43	45	4.89*E* − 12	4
*HSPA8*	11q24.1	cg03309938	2.68*E* − 43	8	0.001265	4
*PCDHGA9*	5q31	cg12648074	3.97*E* − 39	145	6.14*E* − 07	4
*SALL1*	16q12.1	cg01679108	1.39*E* − 38	34	0.000121	4
*TNS1*	2q35	cg18328334	1.23*E* − 37	2	5.00*E* − 19	4
*NPAT*	11q22.3	cg19288979	4.06*E* − 37	26	0.002404	4
*DST*	6p12.1	cg08882472	5.70*E* − 35	38	2.09*E* − 11	4
*CACNA1C*	12p13.33	cg27501686	1.78*E* − 34	77	8.62*E* − 08	4
*ZFHX3*	16q22.2	cg27364780	3.28*E* − 31	45	0.002273	4
*PCDHA1*	5q31.3	cg15122993	7.12*E* − 28	147	1.86*E* − 05	4
*CHD6*	20q12	cg04139300	1.72*E* − 24	2	0.000157	4
*KLHL2*	4q32.3	cg13508949	2.44*E* − 24	9	4.60*E* − 10	4
*EPHA3*	3p11.1	cg16797972	8.13*E* − 23	13	0.001452	4
*ZNF521*	18q11.2	cg14783285	1.54*E* − 17	1	0.001784	4
*DEPDC1*	1p31.3	cg18167921	3.51*E* − 17	5	3.44*E* − 05	4
*PTEN*	10q23.31	cg07263825	2.65*E* − 14	34	3.62*E* − 08	4
*FILIP1*	6q14.1	cg10447080	5.53*E* − 11	1	8.08*E* − 15	4
*SETD5*	3p25.3	cg22811818	3.93*E* − 10	5	0.030099	4
*TLK1*	2q31.1	cg24772525	3.23*E* − 09	7	8.50*E* − 06	4
*COL11A1*	1p21.1	cg26913669	9.30*E* − 09	7	0.000893	4
*SMAD4*	18q21.2	cg10315128	7.56*E* − 06	1	0.000186	4
*CSMD1*	8p23.2	cg12258042	0.000152	1	0.046321	4
*ZFPM2*	8q23	cg17154315	0.004447	1	1.41*E* − 11	4
*MTOR*	1p36.22	cg03956606	0.004852	1	0.000207	4
*TBC1D2*	9q22.33	cg13732677	0.020917	1	1.48*E* − 14	4

**Table 3:** List of the 41 genes containing germline, somatic, and epigenetic mutations transcriptionally associated with aggressive PCa discovered through analysis and integration of multi-omics data.

Genes	GWAS		Methyl P-values			RNA-Seq	
	SNP_ID	*P* value	Probes	*P* value	CpG sites	*P* value	Somatic mutation
*ADNP*	rs12480328	5.00*E* − 11	cg13940160	1.56*E* − 19	10	0.000235	1
*AMIGO2*	rs5759167	2.00*E* − 06	cg08135379	1.71*E* − 15	9	1.78*E* − 05	1
*ANK2*	rs7694725	2.00*E* − 06	cg16931969	4.77*E* − 27	31	4.78*E* − 15	1
*ATF7IP*	rs3213764	2.00*E* − 09	cg00236831	0.026384	1	0.001392	3
*B3GAT1*	rs878987	5.00*E* − 08	cg22777979	1.80*E* − 29	15	4.02*E* − 12	1
*BCL11A*	rs2556375	6.00*E* − 19	cg01616628	5.31*E* − 15	28	9.29*E* − 11	2
*BRCA1*	rs1799950	0.01	cg19531713	2.65*E* − 32	25	0.015169	1
*CDYL*	rs79774606	9.00*E* − 06	cg08249424	9.85*E* − 30	29	0.011624	1
*COL6A3*	rs7584330	3.00*E* − 09	cg00779216	2.82*E* − 28	43	0.000158	2
*DNAH5*	rs887391	2.00*E* − 06	cg05149258	1.61*E* − 26	1	4.17*E* − 25	2
*EPHA10*	rs731174	5.00*E* − 06	cg01967642	1.07*E* − 32	15	3.32*E* − 44	1
*FERMT2*	rs8008270	6.00*E* − 16	cg02232988	5.19*E* − 47	11	2.14*E* − 24	1
*FGFR2*	rs10886902	2.00*E* − 53	cg18566515	1.31*E* − 38	42	1.83*E* − 20	1
*FTO*	rs9939609	0.04	cg12495954	5.00*E* − 38	13	6.25*E* − 11	1
*HAPLN1*	rs4466137	3.00*E* − 06	cg18343881	2.18*E* − 11	3	0.011965	1
*ITGA6*	rs12621278	2.00*E* − 42	cg24530074	6.79*E* − 40	22	4.86*E* − 06	1
*KCNN3*	rs1218582	1.00*E* − 08	cg12058501	1.32*E* − 13	11	1.06*E* − 05	1
*KIAA1211*	rs629242	7.25*E* − 07	cg12879013	1.75*E* − 09	1	0.006964	2
*KIF13A*	rs10456809	5.00*E* − 06	cg09723635	1.07*E* − 39	17	0.041976	3
*KLK2*	rs2735839	6.00*E* − 37	cg05935086	2.92*E* − 43	5	1.49*E* − 14	1
*KLK3*	rs17632542	2.00*E* − 34	cg17687962	4.00*E* − 33	5	1.64*E* − 09	1
*LRP1B*	rs10210358	2.00*E* − 06	cg21484213	1.17*E* − 09	5	1.11*E* − 17	10
*MDM4*	rs4245739	3.00*E* − 24	cg20286844	9.68*E* − 17	6	0.000471	2
*MYO9B*	rs11666569	8.00*E* − 09	cg24679890	7.85*E* − 43	21	0.005102	1
*NOTCH4*	rs3096702	1.00*E* − 11	cg11753286	7.37*E* − 35	29	0.000522	1
*OTX1*	rs58235267	6.00*E* − 07	cg11935853	0.002988	1	5.46*E* − 24	1
*PDLIM5*	rs17021918	1.00*E* − 24	cg09885664	9.66*E* − 20	18	2.99*E* − 20	1
*PHF20L1*	rs2472537	0.000212	cg27342122	3.77*E* − 13	5	0.036862	1
*PKNOX2*	rs138466039	2.00*E* − 11	cg22956116	6.90*E* − 41	2	0.002475	1
*POU2F2*	rs61088131	9.00*E* − 09	cg07716663	1.75*E* − 05	1	4.03*E* − 09	1
*PRDM15*	rs6586243	7.79*E* − 06	cg06555093	1.20*E* − 22	22	5.44*E* − 12	2
*SLC19A2*	rs3765227	0.000126	cg00893538	5.84*E* − 11	2	3.62*E* − 12	1
*SMAD9*	rs140971918	4.00*E* − 06	cg03283486	3.35*E* − 24	13	1.45*E* − 06	1
*TBX1*	rs2238776	2.00*E* − 08	cg24753662	7.76*E* − 20	26	4.61*E* − 08	1
*TBX3*	rs11067228	1.00*E* − 14	cg06211872	2.47*E* − 08	7	0.001996	2
*TBX5*	rs1270884	1.00*E* − 18	cg25556579	4.59*E* − 24	40	1.49*E* − 05	1
*TCF4*	rs28607662	3.00*E* − 08	cg00657460	5.45*E* − 33	11	1.35*E* − 07	1
*TCF7L2*	rs7094871	5.00*E* − 08	cg10983115	7.26*E* − 31	37	2.96*E* − 05	2
*TTC7A*	rs10194115	5.00*E* − 07	cg04574383	1.24*E* − 14	5	3.66*E* − 10	1
*VGLL3*	rs9757252	5.00*E* − 06	cg16373010	1.29*E* − 08	1	0.003787	1
*ZNF652*	rs7210100	3.00*E* − 13	cg07164631	5.54*E* − 14	9	3.89*E* − 07	1

## Data Availability

Original clinical information, mutation, gene expression, and DNA methylation data used in this study were downloaded from The Cancer Genome Atlas (TCGA) via the Genomics Data Commons and are available at https://www.cancer.gov/about-nci/organization/ccg/research/structural-genomics/tcga via the GDC https://gdc.cancer.gov/. Germline mutations were derived from the literature (Supplementary Table SA) and from the Genome-wide Association Information (GWAS) catalog located at the NHGRI-EBI Catalog of published genome-wide association studies data at https://www.ebi.ac.uk/gwas/downloads/summary-statistics. Additional data is shared through supplementary tables referenced in the manuscript and listed in the manuscript and provided as supplementary material to this report.

## Supplementary Materials

Supplementary 1 Table S1: a comprehensive list of single-nucleotide polymorphisms (herein called genetic variants) and associated genes associated with the increased risk of developing prostate cancer and published GWAS reports denoted by the PubMed ID and actual reference from which the data was extracted.

Supplementary 2 Table S2: a comprehensive list of somatic mutated genes and number of somatic mutation events in Aggressive PCa.

Supplementary 3 Table S3: a complete list of all significantly differentially methylated genes distinguishing tumour samples from controls along with the number of differentially methylated CpG sites.

Supplementary 4 Table S4: a complete list of all the significantly differentially expressed genes distinguishing tumour samples from controls along with estimates of P-values and LogFC.

Supplementary 5 Table S5: a complete list of all 6,486 differentially methylated genes which were also differentially expressed along with *P* values and CpG sites.

Supplementary 6 Table S6: a complete list of all the 1702 genes containing somatic, epigenetic and gene expression variation associated with aggressive PCa.

Supplementary 7 Table S7: a complete list of the 796 somatic mutated genes transcriptionally associated to aggressive PCa and a complete list of the 1,264 somatic mutated genes aberrantly methylated in aggressive PCa.
